# Intraspinal choristoma in the lumbar region: A case report

**DOI:** 10.1097/MD.0000000000029350

**Published:** 2022-09-16

**Authors:** Jinxin Yang, Qianlei Liang, Yan Wang, Liang Han, Yongchuan Guo

**Affiliations:** a Departments of Neurosurgery, China-Japan Union Hospital, Jilin University, Changchun, China; b Departments of Pathology, China-Japan Union Hospital, Jilin University, Changchun, China.

**Keywords:** choristoma, congenital intraspinal tumours, intraspinal choristoma

## Abstract

Intraspinal choristoma is a relatively uncommon intervertebral canal tumor. Prior to our reports, only 2 cases of intraspinal choristoma had been reported. Because this disease is not common and looks like a mass of fatty tissue on the magnetic resonance imaging (MRI), intraspinal choristoma can be easily misdiagnosed as teratomas or lipomas (like the case of this article presenting) without a pathology report. So if a lumber intraspinal lesion is discovered in a clinical examination, intraspinal choristoma should be considered as a differential diagnosis. We present a case of intraspinal choristoma that is unlike any other reported case.

**Patient concerns:**

A 35-year-old woman with left lower extremity hypoesthesia and burning-like pain in the lumbar region for 1 month visited the local hospital for plain lumbar spine MRI. The patient was diagnosed with a lumbar space-occupying lesion. A second plain lumbar spine MRI scan and a MRI scan with enhancement were performed in our hospital to confirm the presence of a congenital lipoma in the spinal canal. A postoperative biopsy of the lumbar spinal mass indicated that the mass was an intraspinal choristoma located in the spinal canal.

**Diagnosis:**

Intraspinal choristoma.

**Intervention:**

The lesion was surgically removed, and follow-up plain and enhanced MRI images of the patient’s lumbar spine were obtained.

**Outcomes:**

After surgery, the patient no longer experienced the burning pain in her lumbar region or the left lower extremity hypoesthesia when the patient was discharged. And there was no evidence of recurrence 2 years after the surgery.

**Lessons:**

The MRI presentation of intraspinal choristoma is similar to intraspinal lipoma. Therefore, a pathological assessment is critical to provide an accurate diagnosis.

## 1. Introduction

Choristoma is a tumor-like structure composed of normal tissue located at an abnormal site.^[1]^ While it can occur anywhere in the body, it is more frequently observed in the head and neck region, especially in the pituitary gland^[2]^ and oral cavity.^[3]^ Intraspinal choristomas are rare and, to the best of our knowledge, only 2 cases have been reported.^[4,5]^ Thus, it is pretty rare for choristoma to occur in the spinal canal. The postoperative pathological assessment of an intravertebral occupying lesion in our patient showed a case of intraspinal choristoma. In addition, this patient also had a combination of spinal cord deformities such as spinal cord tethering syndrome which makes this case unique.

## 2. Case Report

### 2.1. Clinical summary

A 35-year-old woman was diagnosed with an intraspinal space-occupying lesion after a magnetic resonance imaging (MRI) scan of her lumbar spine was performed at a local hospital. The presenting complaint was hyperalgesia of the left lower extremity and burning pain in the lumbar region for 1 month. The patient was admitted to our hospital for further diagnosis and treatment. The preoperative physical examination revealed scoliosis, pain elicited with positive pressure on both sides of the fourth lumbar vertebra, and bilaterally diminished Achilles tendon and knee tendon reflexes. The straight-leg raising test was positive. The extension-hallucis test was positive, and the tension test for the femoral nerve was negative.

**Figure 1.**
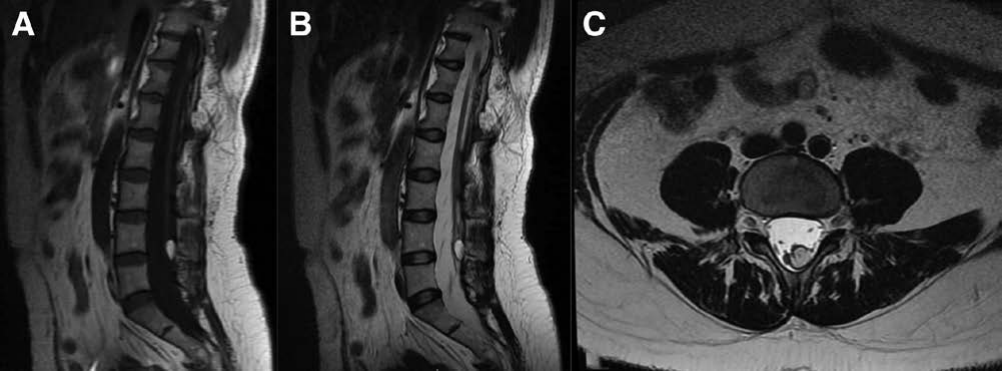
(A) The preoperative lumbar spine plain MRI scan using a T1 sequence in the sagittal plane. (B) The preoperative lumbar spine plain MRI scan using a T2 sequence in the sagittal plane. (C) The preoperative lumbar spine plane MRI scan using a T2 sequence in cross-section. MRI = magnetic resonance imaging.

**Figure 2.**
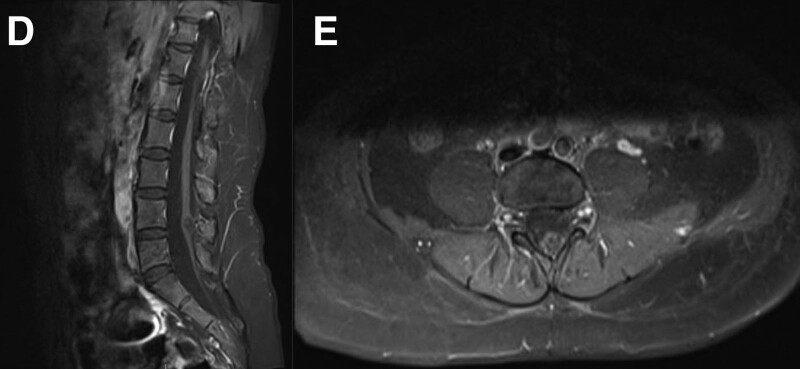
(D and E) Preoperative lumbar spine enhanced MRI scans in the sagittal plane and cross-section, respectively. MRI = magnetic resonance imaging.

MRI of the lumbar spine revealed that a near-circular short T1 and a long T2 signal shadow with clear borders were observed at the level of the inferior border of the fourth lumbar vertebral body. A low signal of Short Time Of Inversion Recovery demonstrated a rounded, non-enhanced area at the level of the lower edge of the fourth lumbar vertebral body. The area exhibited clear borders and was approximately 1.0 cm in diameter within the spinal canal (Figs. 1 and 2). In addition to intraspinal choristoma, other spinal cord deformities were observed on the sagittal MRI images in this patient: the spinal cord extended to the level of the lower edge of the fourth lumbar vertebra, which was distinctly abnormal because the normal adult spinal cord does not extend beyond the lower edge of the first lumbar vertebra, and the spinal cord was irregularly divided into 2 parts beginning at the level of the lower border of the 11th thoracic vertebral body, ending at the lower border of the fourth lumbar vertebra body. These images indicated the presence of a tethered spinal cord, resulting in shortened spinal cord end filaments. Furthermore, the corresponding region of the spinal canal and the subarachnoid space were widened significantly, suggesting the presence of a possible developmental abnormality associated with the spinal canal. Therefore, this patient also was diagnosed with tethered spinal cord syndrome and spinal cord deformities.

**Figure 3.**
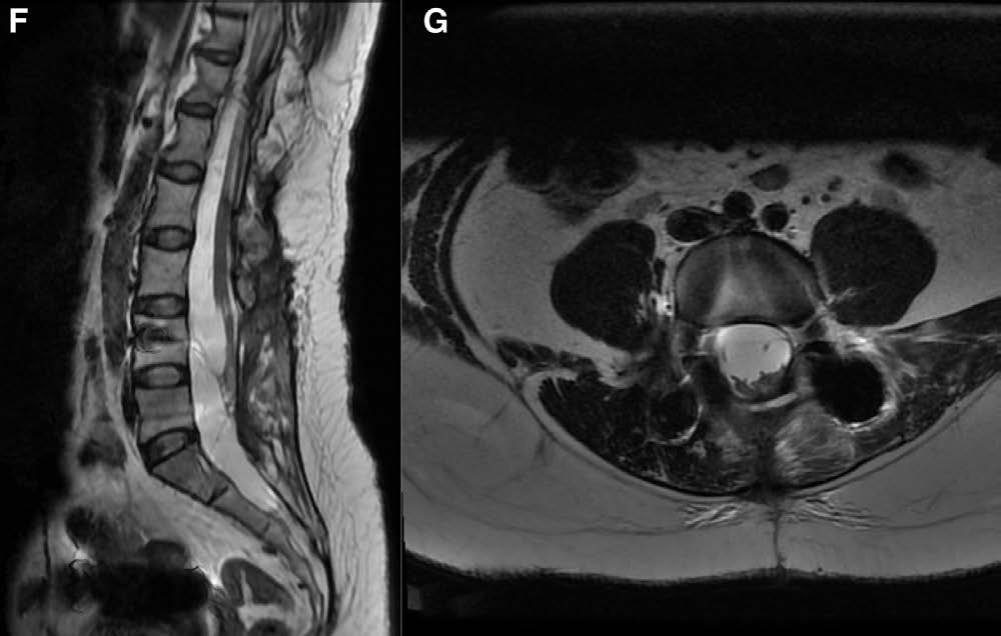
(F and G) Images taken 2 y after the surgery. The lumbar spine plain MRI scan in sagittal section (F) and cross-section (G). MRI = magnetic resonance imaging.

The pre-surgical Japanese Orthopedic Association Score (JOA score) was 14. Preoperatively, the lesion was thought to be a lumbar intraspinal lipoma. The mass was surgically removed under general anesthesia. During the surgery, the tumor appeared yellow and exhibited a tough texture. The patient’s burning pain in her lumbar region and the left lower extremity hypoesthesia disappeared after surgery. One month after surgery, the physical examination revealed that pain was not elicited with positive pressure on both sides of the fourth lumbar vertebra. The straight-leg raising test was positive, and the extension-hallucis test was negative. Furthermore, the bilateral Achilles tendon and knee tendon reflexes had returned to normal. The JOA score was 27 at discharge, and the improvement rate was 86.7%. We conducted a 2-year follow-up examination, and the JOA score was 28 in the second year after surgery, and the patient’s improvement rate was 93.3%. An MRI scan was taken 2 years after the surgery, and there was no evidence of recurrence (Fig. 3).

### 2.2. Pathological findings

**Figure 4.**
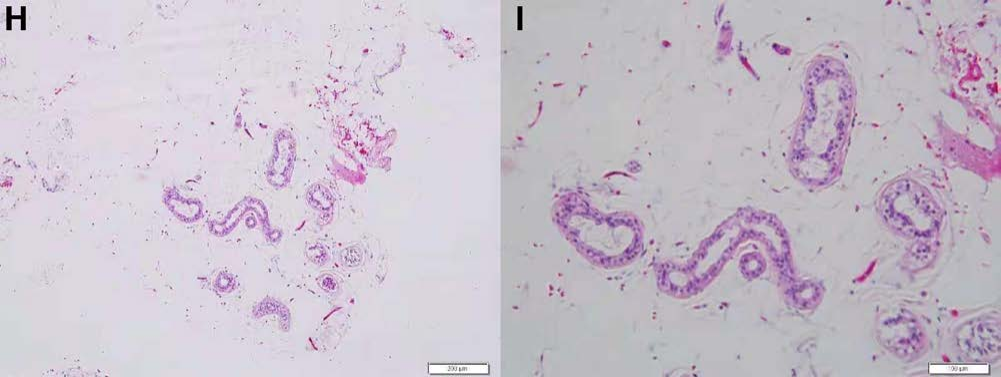
(H) Microscopic image of the tumor tissue with H&E staining (×100 magnification). (I) Another microscopic image at a magnification of ×200. (H and I) Mature fibrous and adipose tissue, blood vessels, epithelium, small sweat gland ducts, and a small amount of squamous-like tissue.

**Figure 5.**
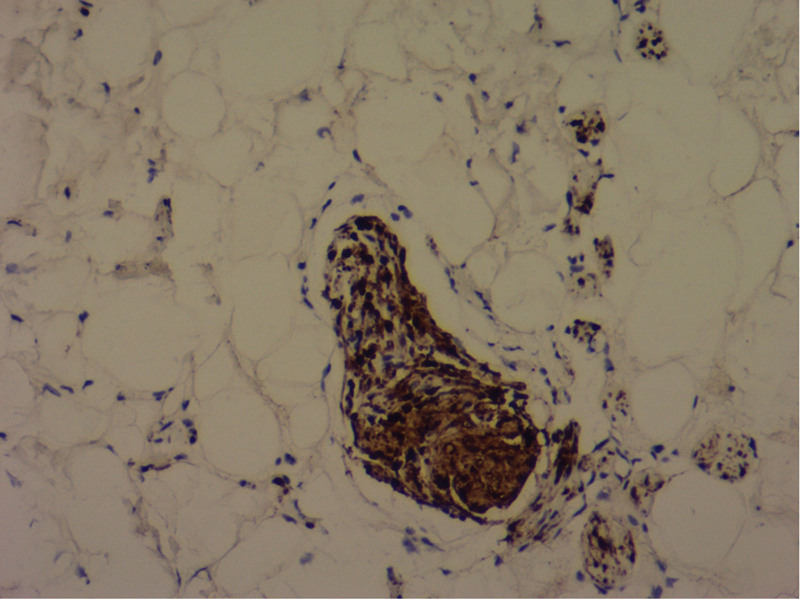
Immunohistochemical staining for S-100 protein (H&E ×100).

**Figure 6.**
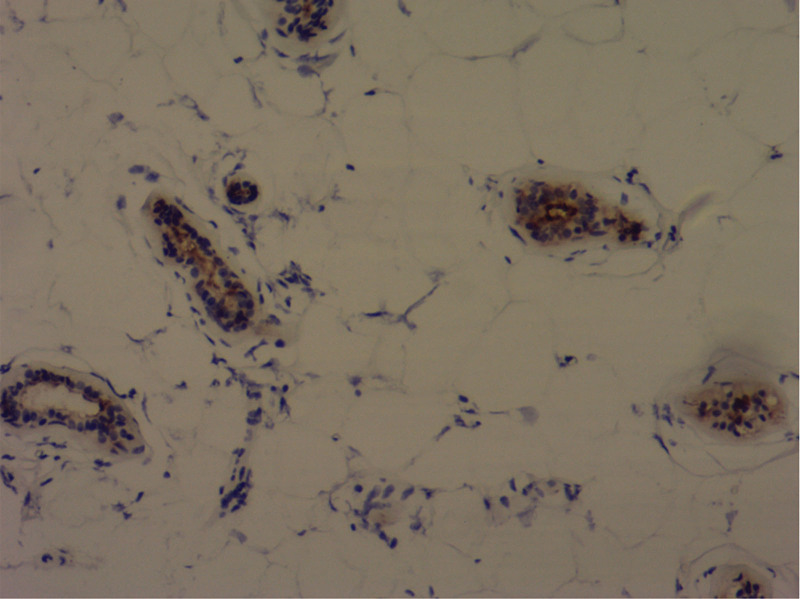
Immunohistochemical staining for GCDFP-15 protein (H&E ×100).

**Figure 7.**
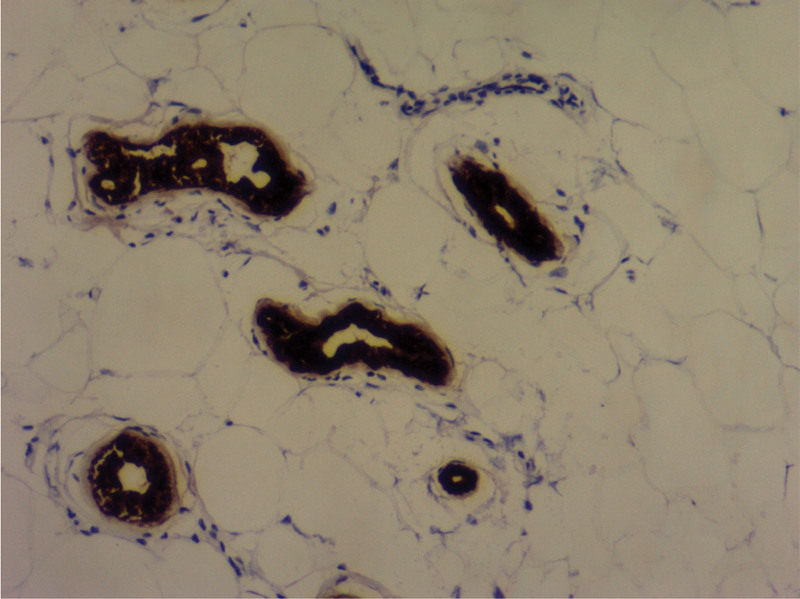
Immunohistochemical staining for CK7 protein (H&E ×100).

**Figure 8.**
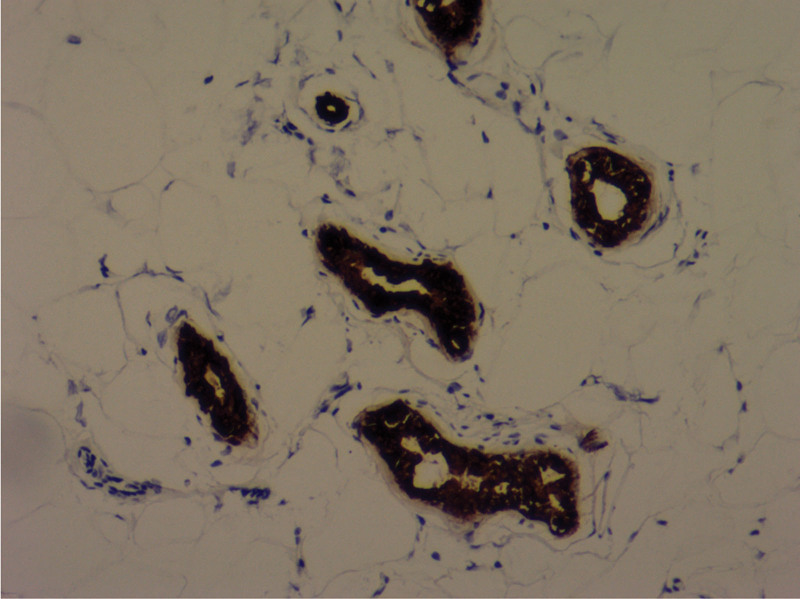
Immunohistochemical staining for CK8 protein (H&E ×100).

**Figure 9.**
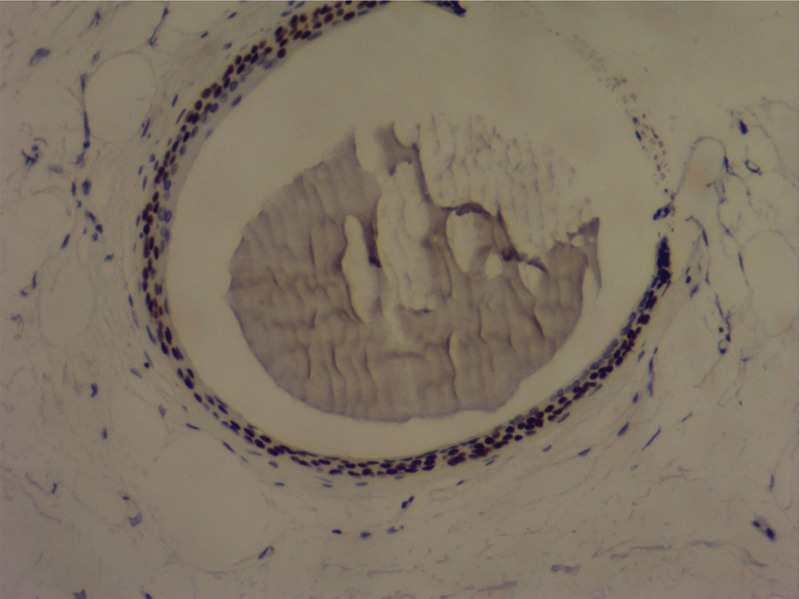
Immunohistochemical staining for P63 protein (H&E ×100).

**Figure 10.**
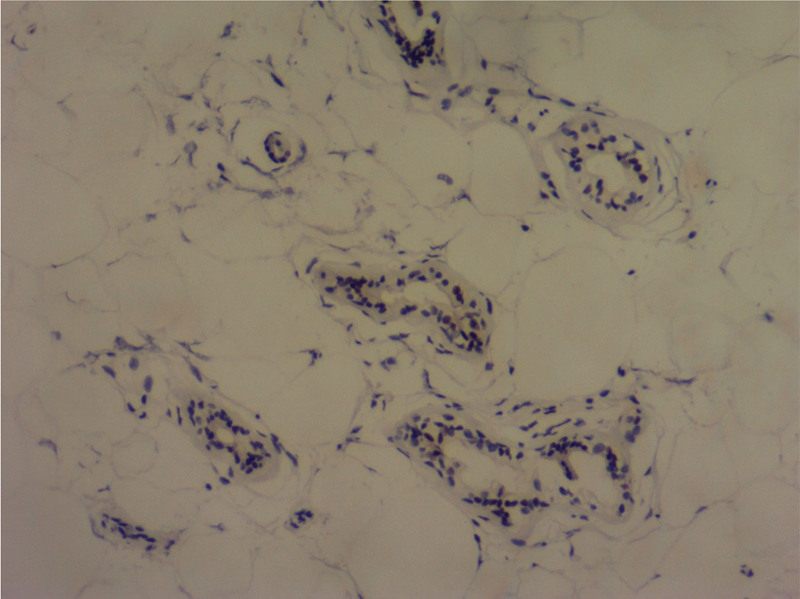
Immunohistochemical staining for AR protein (H&E ×100).

The histological examination provided the following information: the tumor size was 1.5 cm × 1 cm × 0.4 cm; the color was primarily gray to yellow; the appearance was nodule-like, soft, with a slightly tough localized region of gray-white color. A small amount of bone tissue was present. The microscopic examination revealed the following information: mature fibrous and fatty tissues, blood vessels, and epithelium were present; small sweat gland ducts also were seen. The histological pattern resembled an intraspinal choristoma (Fig. 4). Immunohistochemical features were assessed based on immunohistochemical staining, including positive staining for S-100 (Fig. 5), GCDFP-15 (Fig. 6), CK7 (Fig. 7), CK8 (Fig. 8), P63 (Fig. 9), and AR (Fig. 10). We summarized the comparison of results of the immunohistochemical features of choristoma and hamartoma in Table 1.^[4,6–17]^

**Table 1:** Summary of immunohistochemical features of reported choristoma and hamartoma cases in the literature.

Diagnosis	Phakomatous choristoma①	Phakomatous choristoma②	Cartilaginous choristoma	Lacrimal gland choristoma	Palatal osseous choristoma	Mammary choristoma	Intraspinal choristoma	Pancreatic endocrine heterotopia	Leiomyomatous hamartoma	Hamartoma of mature cardiac myocytes	Pulmonary hamartoma	Schwann cell hamartoma	Sinonasal seromucinous hamartoma
Reference	S D et al^[6]^	SM R et al^[7]^	GW Pereira et al^[8]^	A T-E et al^[9]^	R S, et al^[10]^	Chen X, et al^[11]^	PF C, et al^[4]^	M S, et al^[12]^	Zhang M, et al^[13]^	JC M-H, et al^[14]^	Rossi G^[15]^	Li Y, et al^[16]^	YW H, et al^[17]^
S-100	Positive	Positive	Positive		Negative		Positive		Positive			Positive	Some cases positive
EMA	Negative	Negative		Positive	Negative							Negative	Some cases positive
desmin									Positive	Positive	Positive	Negative	Some cases positive
AE1/AE3			Negative	Positive				Positive		Negative			
SMA		Negative		Positive					Positive		Positive	Negative (α-SMA)	Some cases positive
CD34		Negative								Positive		Negative	
p40											Negative		
p63			Negative			Positive					Negative		Focal positivity
Synaptophysin		Negative						Positive					
Cytokeratin	Negative	Negative			Negative								
Synaptophysin		Negative						Positive					
Cytokeratin	Negative	Negative			Negative								
CK5/6											Negative		
CK7				Positive				Positive			Positive		Positive
Vimentin	Positive	Positive					Negative						
Glial fibrillary acidic protein	Negative	Negative					Negative						
Melan-A		Negative											
CK19								Positive					Positive
CAM5.2				Positive									
NSE				Positive									
CK20				Negative							Negative		Negative
Actin muscle-specific antibody										Positive			
Calretinin										Negative	Negative		
Diagnosis	Phakomatous choristoma①	Phakomatous choristoma②	Cartilaginous choristoma	Lacrimal gland choristoma	Palatal osseous choristoma	Mammary choristoma	Intraspinal choristoma	Pancreatic endocrine heterotopia	Leiomyomatous hamartoma	Hamartoma of mature cardiac myocytes	Pulmonary hamartoma	Schwann cell hamartoma	Sinonasal seromucinous hamartoma
Reference	S D et al^[6]^	SM R et al^[7]^	GW Pereira et al^[8]^	A T-E et al^[9]^	*R* S et al^[10]^	Chen X, et al^[11]^	PF C, et al^[4]^	DJ R et al^[12]^	Zhang M et al^[13]^	JC M-H et al^[14]^	Rossi G et al^[15]^	Li Y, et al^[16]^	YW H et al^[17]^
Calretinin										Negative	Negative		
High molecular weight keratin (HMWK)													Positive
Islet-1								Positive					
CDX2								Positive			Negative		
CK27								Negative					
TTF-1 (SPT24)								Negative			Negative		
Trypsin								Negative					
Insulin								Positive					
Glucagon								Positive					
Somatostatin								Positive					
CD31								Negative					
ERG								Negative					
Diagnosis	Phakomatous choristoma①	Phakomatous choristoma②	Cartilaginous choristoma	Lacrimal gland choristoma	Palatal osseous choristoma	Mammary choristoma	Intraspinal choristoma	Pancreatic endocrine heterotopia	Leiomyomatous hamartoma	Hamartoma of mature cardiac myocytes	Pulmonary hamartoma	Schwann cell hamartoma	Sinonasal seromucinous hamartoma
Reference	Kurman et al^[6]^	Seyed et al^[7]^	GW P et al^[8]^	A T-E et al^[10]^	R S, et al^[9]^	Chen et al^[11]^	PF C, et al^[1]^	M S, et al^[17]^	M Z, et al^[13]^	JC M-H, et al^[12]^	G R^[15]^	Y L et al^[14]^	YW H, et al^[17]^
Pan-CKs											Positive		
MUC5AC											Positive		
Napsin A											Negative		
MUC1											Negative		
MUC2											Negative		
MUC6											Negative		
CD7													Positive
CD19													Positive
Proliferating cell nuclear antigen													Positive
Estrogen receptor						Positive							
Progesterone receptor						Positive							
Ki67						Positive							
CEA							Positive						
CK (glandular)							Positive						
Neurofilament protein							Positive						
Claudin-1												Negative	
Glucose transporter-1												Negative	
Melan-A												Negative	
HMB-45												Negative	
c-Kit												Negative	
Chromogranin A								Positive					

EMA = epithelial membrane antigen, HMWK = high molecular weight keratin, NSE = neuron-specific enolase, SMA = smooth muscle actin.

## 3. Discussion

Based on the pathological diagnosis, the features of choristoma and hamartoma can be confounding. Both are rare, and many clinicians and pathologists find it challenging to identify them accurately. In addition, many articles have mentioned the need to be able to differentiate between choristoma and hamartoma. However, none of the previous reports have clarified the critical features that facilitate differentiation. The term choristoma is derived from the Greek root “choristos,” meaning separated and also means mass or tumor. The term is synonymous with heterotopia and ectopia, which are defined as the proliferation of normal cells in an abnormal location^[1]^ and an ectopic, histologically normal mature tissue.^[18]^ Hamartoma is defined as a lesion containing mature and well-differentiated ectoderm and mesoderm derivatives that appear in abnormal locations^[2,19]^; usually, 1 component dominates.^[2]^ Hamartoma is similar to choristoma in that, both are benign proliferations of mature tissue, but a hamartoma is a malformation resembling the tissue at its site of origin, while the choristoma contains components that do not usually appear at the anatomic location where it is located. Therefore, choristoma can be considered to be a subtype of hamartoma.^[20]^ Thus, a “hamartoma” confirmed histologically to contain ectopic tissue is actually a choristoma.^[21]^ Castillo et al noted in an article on midline spinal cord hamartomas that only hamartomas that contained local elements in a disorganized fashion could be considered pure hamartomas, while those with normal but ectopic tissues (including glands, lymphoid tissue, and urinary tract tissues) should be considered choristomas.^[21,22]^ Malela et al^[19]^ reported a sporadic case of “Congenital Midline Spinal Hamartoma in a 5-Month-Old Infant in 2020.” The similarity with our case is that it also contained numerous adipocytes, however, the case reported by Malela et al did not exhibit cartilage, skeletal muscle, mature collagen fibers, nerve and vascular components in the normal tissue at the location of the choristoma. The histologic examination of our case revealed mature fibrous tissue and squamous epithelium, as well as small sweat gland ducts. These 3 mature tissue components should not be present in the spinal canal, so the histologic examination indicated that a choristoma was present in the patient in this report.

Since choristoma is extremely rare, it is not usually suspected before the surgery and pathology. Alternative differential diagnoses include teratoma and intraspinal lipoma. It was noted that the preoperative imaging presentation of this patient was similar to intraspinal lipoma. Teratomas contain cell populations from at least 2 of the 3 primary germ layers, endoderm, mesoderm, and ectoderm. They form during embryonic development and may be diagnosed at any age. Teratomas are thought to result from the failure of primitive germ cells to migrate and usually occur along the body midline, anywhere from the coccyx to the pineal gland. The primitive germ cells fail to degenerate and subsequently proliferate in abnormal locations in the midline to form tumors. Thus, the pluripotent capacity of these cells gives rise to multiple tissues consisting of immature cells that have the potential for malignant degeneration.^[23]^ There are 4 histological teratoma variants: mature teratoma, immature teratoma, malignant teratoma, and monodermal teratoma.^[23]^

Intraspinal lipoma also is a rare benign tumor, accounting for <1% of all spinal cord tumors.^[22]^ Lesions often are located in the lumbosacral region, while occurrence within the dura of the cervical and thoracic medulla is less common. Though the pathogenesis of intradural lipomas remains unclear but occasional association with other midline anomalies points to a developmental origin.^[24]^ Intraspinal lipomas often occur in conjunction with a tethered spinal cord, and the lesion may contain other types of tissues such as bone, cartilage, or even bone marrow. Ammerman et al^[25]^ had described the clinicopathological characteristics of intramedullary lipomas: the tumors tend to be subpial in origin, involve multiple spinal cord segments as well as adjacent nerve roots, and have no clear plane demarcating themselves from adjacent neural parenchyma. Histologic investigation classically demonstrates normal adipose cells with intervening collagen. The collagen is often in continuity with adjacent coverings of vessels and neural structures.^[26]^ As no clear cleavage plane is found between the intraspinal lipoma and spinal cord, very few reports recommend complete surgical removal of an intraspinal lipoma as complete resection is associated with significant postoperative complications.^[27]^ Our literature review revealed that patients with intraspinal lipoma have masses that exhibit an irregular morphology and no clear boundary with the spinal cord on preoperative MRI scans. Whereas in this case, the tissue mass presented a more round shape and a more distinct boundary. These characteristics might help differentiate choristomas from intraspinal lipomas using MRI in the future. These observations also suggest that surgery for choristomas differ from lipomas in that choristomas can be less aggressive, be completely resected, and have a predictably lower postoperative recurrence rate than intraspinal lipomas. In contrast, intraspinal lipomas are more closely related to peripheral nerve tissue. It is possible to reduce the size of an intraspinal lipoma by reducing the amount of body fat, which would alleviate the symptoms of spinal cord or cauda equina irritation caused by the tumor. Thus, it is worth considering whether weight loss could be used to reduce the tumor size in patients diagnosed with a suspected choristoma who are unwilling or unable to undergo surgery.

Although microscopic findings can be highly suggestive of cartilaginous choristoma of the tongue, immunohistochemical staining is essential to rule out other possible diagnoses and confirm the hypothesis. For immunohistochemical staining, S-100, GCDFP-15, CK7, CK8, P63, and AR are used as positive indicators for choristoma. Positive GCDFP-15 staining suggested the presence of apocrine sweat glands. Positive staining for CK7 and CK8 suggested epithelial tissue origins, while positive staining for S-100 was indicative that the tumor originated from neurological tissue. P63 is a specific and sensitive marker for human myoepithelial cells, and positive staining suggests that the lesion is more likely to be a benign tumor. Therefore, the results that were obtained confirmed the diagnosis of choristoma.

We also suggest that it was not a coincidence that the patient also exhibited spina bifida and spinal cord tethering, as an association between spinal dysraphism and the occurrence of a lipoma is possible. The etiology of congenital spina bifida is not fully understood. However, most scholars believe that abnormal embryonic development of the neural tube is responsible for spina bifida, and abnormalities in the fusion process result in its formation. Specifically, the neural tube separates from the ectoderm through a process of detachment. After separation, midline fusion of the ectoderm above the neural tube occurs.^[21]^ Besides, a case of autograft-derived spinal cord mass following olfactory mucosal cell transplantation in a spinal cord injury patient,^[23]^ enlightened us that the possible etiology of this intraspinal choristoma is a malformation during the embryonic development, and the malformation results in a transplantation of the regional disordered embryonic cells in lumbar region. Therefore, we believe that the abnormal developmental process of spinal bifida is most likely the reason for the development of intrasipnal choristoma in our case. Based on this case, if a space-occupying lesion in the spinal cord with a predominantly fatty signal appears on the MRI, surgeons should carefully distinguish whether the boundaries between the occupying lesion and the surrounding tissues are distinct. If the lesion has definitive boundaries and the patient has congenital spina bifida or spinal cord embolism, a possible diagnosis of intraspinal choristoma should be considered. Surgical options include complete resection, and the prognosis for patients with intraspinal choristoma is generally considered to have a lower recurrence rate than an intraspinal lipoma.

In general, the clinical course and histopathological features of intraspinal choristoma indicate its benign nature. In 2 reported cases in children, the tumor was incompletely excised. One child was followed for 7.5 years and the other for approximately 16 months, without evidence of recurrence or residual tumor.^[6]^ Because choristoma is classified as a benign tumor, many studies do not discuss recurrence. The case reported in this study showed benign tumor characteristics based on the preoperative MRI findings and the intraoperative observations. We followed the patient in this case for 2 years, and the patient did not exhibit any recurrence, which indicated that surgical excision is curative, with a low incidence of recurrence. However, a case of osseous choristoma that exhibited recurrence was reported in another article, which might have been due to the tumor having a greater degree of malignancy. Typically, tumors that are multiple and have no pedunculated mass surrounded by soft tissue, such as within the buccal mucosa or muscle, might have the possibility of recurrence.^[2]^

## 4. Conclusion

Intraspinal choristoma is a rare congenital intraspinal tumor that might correlate significantly with congenital spinal deformities. Intraspinal choristomas are not easily distinguishable from intraspinal lipomas and intraspinal hamartomas based on MRI. However, if the lesion has distinct boundaries and the patient has congenital spina bifida or spinal cord embolism, a possible diagnosis of intraspinal choristoma should be considered. With the presence of distinct tumor boundaries, surgical options for complete resection should be considered. The prognosis of a lower recurrence rate for an intradural psammoma is generally better than for an intraspinal lipoma.

## Acknowledgments

The authors express their gratitude to all of the staff of the China-Japan Union Hospital of Jilin university who were involved in this patient’s care.

## Author contributions

Resources: Yongchuan Guo, Qianlei Liang,Yan Wang,Liang Han

Supervision: Yongchuan Guo, Qianlei Liang

Writing – original draft: Jinxin Yang

Writing – review & editing: Jinxin Yang
